# Impact of malodour on health‐related quality of life of patients with chronic wounds due to volatile organic compounds

**DOI:** 10.1111/wrr.70033

**Published:** 2025-04-28

**Authors:** E. K. Stuermer, C. Nathrath, H. Lober, M. Wigger, T. M. Janke, M. Augustin, M. Dittmer, S. Sielemann, S. C. Liegenfeld

**Affiliations:** ^1^ Department of Vascular Medicine, Translational Wound Research University Medical Center Hamburg‐Eppendorf (UKE) Hamburg Germany; ^2^ Hamm‐Lippstadt University of Applied Sciences Hamm Germany; ^3^ Institute for Health Services Research in Dermatology and Nursing (IVDP), University Medical Center Hamburg‐Eppendorf (UKE) Hamburg Germany

**Keywords:** bacteria, chronic wounds, HRQoL, ion mobility spectrometry, wound infection, wound odour

## Abstract

Chronic wounds significantly impact health‐related quality of life (HRQoL). Unpleasant wound odour, caused by bacterial colonisation and necrotic processes, is a distressing symptom. Its exact composition is not well understood, but it could be the basis for a personalised odour avoidance strategy. Therefore, this feasibility study explored 92 wounds from 66 patients with the focus on wound odour, bacterial burden and their impact on health related‐quality of life (HRQoL). Volatile organic compounds (VOCs) responsible for wound odours were detected at the molecular level, using gas chromatography–mass spectrometry and ‐ion mobility spectrometry and correlated to the HRQoL. In patients analysed (average age 69 ± 13 years, wound persistence 24.31 ± 70.8 months) 135 pathogens were identified by swabbing including 19% *Staphylococcus aureus*, 15% *Pseudomonas aeruginosa* and 35% *Enterobacteria*. The specific questionnaire ‘Wound‐QoL‐14’ showed a non‐significant difference in HRQoL in patients with wound odour (2.1 ± 1.0 vs. 1.8 ± 1.0). The latter also had the highest number of VOC detections. The most frequently detected, relevant VOCs from prokaryotic sources were dimethyl‐disulphide and diacetyl‐(2,3‐butanedione). Furthermore, potential biomarkers for specific pathogens were identified, including dimethyl‐trisulphide for *P. aeruginosa* and indole for *Escherichia coli*. The most prevalent substance groups were ketones and alcohols. In conclusion, the malodour of chronic wounds is caused by a mixture of the intrinsic odour of bacterial products and necrosis. This exploratory study, which combines the analysis of decoded VOCs and the olfactory assessment of odour, could be a novel, targeted approach for identifying potential ‘anti‐wound odour therapies’ that will significantly benefit the HRQoL of patients with malodourous tumour wounds, in particular.

**Abbreviations**
AI: artificial intelligence
CWC: Comprehensive Wound Center
DFS: diabetic foot syndrome
DMTS: dimethyl trisulphide
e‐nose: electronic nose
FDA: U.S. Food and Drug Administration
G.A.S: Gesellschaft für analytische Sensorsysteme mbH
GC–IMS: gas chromatography–ion mobility spectrometry
GC–MS: gas chromatography–mass spectrometry
HCPs: health care professionals
HRQoL: health‐related quality of life
IMS: ion mobility spectrometer
MS: mass spectrometer
PCR: polymerase chain reaction
PHMB: polyhexamethylene biguanide
VOCs: volatile organic compounds
WHD: wound healing disorder

## 1 INTRODUCTION

Chronic wounds, tumour wounds and necrotizing wounds represent a global health challenge and socio‐economic burden. Around 1.5 to 2 million people in Europe suffer from chronic wounds.^1^ These wounds often require long‐term care, which strains healthcare systems and incurs considerable costs.^2^ The occurrence of complications such as infections reinforces this impact. In addition, chronic wounds significantly impair patients' health‐related quality of life (HRQoL).^2^ Patients often suffer from pain, reduced mobility, social isolation and emotional distress, especially if the wound produces an unpleasant odour, which can lead to feelings of shame and a considerable impairment of general well‐being.

The cause of malodourous wound odour can be attributed to several interrelated factors, including the loss of tissue viability, hypoxia, bacterial colonisation and the metabolic by‐products of bacteria.^3^ Necrotic tissue, in particular, plays a central role in producing unpleasant odours, as it serves as a substrate for bacterial activity.^3^ Various wound dressings and interventions have been developed to address the challenge of wound odour. While these strategies help manage bacterial burden,^4^ their efficacy regarding malodour is often variable, depending on the wound's underlying aetiology (e.g., tumour) and microbial profile (e.g., *Enterobacteria*). Antimicrobial agents such as silver, polyhexamethylene biguanide (PHMB) and iodine are commonly used, as they disrupt bacterial cell walls and interfere with the function of proteins and enzymes essential for bacterial survival.^5^ Sugar or honey inhibits bacterial growth by creating an environment unsuitable for proliferation through osmosis or pH modification.^6^ Activated charcoal acts differently by binding volatile organic compounds (VOCs) released by bacteria, which are the primary contributors to wound odour.^4^ These effects of activated charcoal are already used in some wound dressings.^7^, ^8^, ^9^ Other topical therapies try to mask the wound odour with their inherent odour (e.g., cinnamon) or try to prevent the development of odours by reducing bacterial tissue destruction.^4^, ^10^ In summary, however, there is currently no satisfactory solution in topical therapy to control wound odour, although the existing options contribute to some improvement in HRQoL by reducing odour to a tolerable level—not always, but often.

VOCs are a group of versatile molecules emitted by humans at ambient temperature through skin, breath, wounds and other bodily fluids.^11^ Specific VOCs emitted by bacteria result in the breakdown of metabolic intermediates.^11^ Hydrocarbons, alcohols, aldehydes and organic acids are therefore part of this group. As previously mentioned, they contribute to the unpleasant‐smelling wound odour. Wound odour is usually classified by patients and health care professionals (HCPs) into different odour categories, such as sulphur, sour, cheese, vomit and foot odour. In 2009, Shirasu et al. established that dimethyl‐trisulphide (DMTS), emitted by *Pseudomonas aeruginosa*, is the main cause of the sulphur odour. Identifying and unmasking further VOCs responsible for the wound odour could be a starting point for the development of targeted strategies to compensate or mask this odour so that—in addition to the causal local therapy mentioned above—rapid symptomatic therapy could be provided that directly affects the patient's quality of life.

Patients with wounds whose odour cannot be controlled usually feel uncomfortable and avoid social interactions because they believe they are a burden to others. They may even delay necessary medical treatment out of shame.^3^ However, not only do the patients suffer from the unpleasant wound odour, but also relatives and health care professionals who are confronted with the smell and may even develop disgust. They must develop coping strategies to manage these situations without letting the patient know how difficult it is for them.^12^ Given the still unresolved issue of malodours, this study aims to explore the complex interplay between the microbiological, biochemical and sensory aspects of wound odour to improve current management strategies focusing on and enhancing the quality of life for patients suffering from malodorous wounds. It provides a comprehensive framework for understanding the multi‐faceted nature of malodourous wounds and tries to pave the way for more effective treatment strategies. These biochemical analyses of wound odour deliberately address ‘only’ one symptom, but one that is high on the list of social and psychological impairments for patients who suffer from it.

## 2 METHODS

### 2.1 Study design, patients and clinical assessment

Patients suffering from (chronic) wounds were recruited between January and September 2024 at a Comprehensive Wound Center (CWC) in Europe. Written informed consent was obtained from patients for wound odour sample collection and capture of wound pictures. During the study visit, demographic data, the underlying diseases, other pre‐existing conditions, (long‐term) medication, a detailed wound assessment (wound size, description of wound coatings and exudation, photo documentation of the wound) and potential nicotine abuse were documented.

Health care professionals rated the intensity of wound odour on a scale of 0–10 (none to very strong). If the bacterial load of the wound was unknown, a wound swab was taken using the Essen circle technique.^13^ For patients with multiple wounds and a sufficient distance between them, for example, on the right and left feet or lower leg, each was chosen for odour collection. Specific inclusion criteria other than the presence of a (chronic) wound were not defined. Exclusion criteria related to lack of informed consent, Alzheimer's disease or vascular dementia and pregnancy.

The study was approved by the Ethics Committee of the Hamburg Medical Association (PV5883‐4183‐BO‐ff). It was therefore conducted in accordance with the ethical standards laid down in the vote of August 2018 and its subsequent amendments (last amendment 29 August 2024).

### 2.2 Assessment of HRQoL

Before dressing change and wound odour collection, patients filled in a comprehensive questionnaire addressing the HRQoL of people with chronic wounds, the Wound‐QoL‐14.^14^, ^15^ The questionnaire consists of 14 items on impairment in patient's HRQoL. The items are answered on a 5‐point Likert scale (0 ‘not at all’ to 4 ‘very’). A total score can be calculated (0–4) with higher scores indicating higher impairments. In addition to the comprehensive assessment of all Wound‐QoL‐14 data, we analysed the HRQoL outcome in the subgroups ‘no to low wound odour’ and ‘moderate to high wound odour’. To elaborate on associations between the HCP assessment of wound odour and patients' HRQoL, we conducted Spearman correlations between the HCP assessment and the Wound‐QoL‐14 total score as well as between the HCP assessment and the odour‐related Wound‐QoL‐14 item.

### 2.3 Assessment of patient‐reported outcomes

In parallel, demographic data, the reason for the wound or the underlying disease and other relevant pre‐existing conditions were documented. After collecting wound odour samples (see next paragraph) a detailed wound assessment (wound size, description of wound coatings and exudation, photo documentation of the wound), local wound therapy and quantification of wound pain by VAS score were performed.

### 2.4 Sampling of wound odour

**FIGURE 1:**
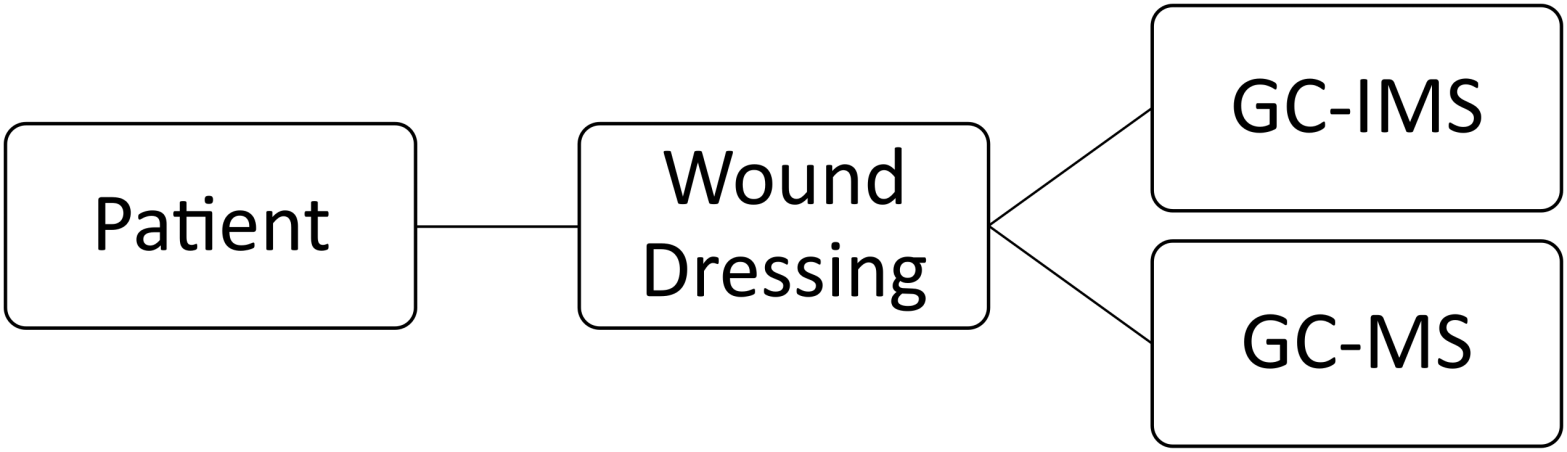
Overview of the samples taken with the instrumental analysis conducted (GC–MS, gas chromatography–mass spectrometry; GC–IMS, gas chromatography–ion mobility spectrometry).

To analyse the VOCs responsible for the wound odour, the wound dressing was divided into two pieces, each measuring 2 cm by 5 cm, and then immediately sealed within a 20 mL glass headspace vial. Samples of the ambient air within the treatment room were also collected as a reference. Furthermore, samples of unused dressings commonly used in the treatment process, including superabsorber and foam dressings, were taken. The vials were transported to the Hamm‐Lippstadt University of Applied Sciences laboratory, where they were analysed. A summary of the analysis is provided in Figure 1. The study protocol ensured consistent sampling conditions and maximised the reliability of the collected data.

### 2.5 Analysis of wound odour

The sealed vials containing the wound dressing were processed in an autosampler, specifically the AOC‐5000 Plus (Shimadzu Corporation, Kyoto, Japan). The autosampler was shaking and tempering (80°C, 20 min) the vials. An airtight syringe injected the headspace sample (2 mL) into the GCMS‐QP2010 Ultra (Shimadzu Corporation), which had two columns: one leading to the mass spectrometer (MS) and the other to the ion mobility spectrometer (IMS; G.A.S Gesellschaft für analytische Sensorsysteme mbH, Dortmund, Germany).

The two injection ports were maintained at a temperature of 150°C and had a split ratio of 1:5. Both columns (HP‐5 MS UI 30 m × 0.25 mm × 0.5 μm, Agilent, Santa Clara, CA, USA) were operated at a constant flow rate of 35 cm/sec (approximately 0.93 mL/min) with helium as the carrier gas. Moreover, a temperature ramp was used, maintaining a temperature of 50°C for 4 min, increasing by 5°C per minute to 150°C, by 10°C per minute to 200°C and then holding it for a total measurement time of 35 min. The mass spectrometer was maintained at an ion source temperature of 200°C with 70 eV electron energy and a scanning range of *m/z* 32–300. The ion mobility spectrometer contained a drift tube (15.2 × 53 mm) heated to 80°C, which utilised nitrogen as the drift gas. Ionisation was done by atmospheric pressure chemical ionisation through a tritium source.

### 2.6 Identification and processing of VOCs

The IMS data are presented in the VOCal software (G.A.S. mbH) as a heat map, which illustrates the GC runtime (retention time) and the drift time in relation to the reactant ion peak. To identify substances in GC–IMS, it is necessary to measure reference substances, which is very laborious and can only be done for known substances. For the screening of unknowns, it was necessary to use a GC–MS additionally, which allows the identification of compounds via database comparison. The GC–MS measurements were processed using the software GCMS Solutions (version 4.52, Shimadzu Corporation). The identification of substances by the mass spectra was conducted using the NIST library (NIST/EPA/NIH Mass Spectral Library 14, National Institute of Standards and Technology of the U.S. Department of Commerce). Due to a retention time shift in the data of GC–MS and GC–IMS, the signals were correlated using retention indices^16^ based on a measured ketone mixture.^17^

After identifying the substances and correlating them to the GC–IMS signals in VOCal, the list of substances and their intensity (in mV) was exported and processed in Excel. The final output is a table displaying the frequencies of substances and the samples in which they occurred.

It should be noted that there are numerous additional sources of volatile organic compounds in the surrounding environment. Consequently, it was necessary to use blank samples of the surrounding area, wound dressings and other products applied directly to the wound to obtain accurate measurements (Table 1).

**TABLE 1:** Summary of the tested products to determine their VOCs.

Type	Manufacturer	Product/brand name
Paraffin gauze dressing	Urgo Medical	Urgotül
Multi‐layered bordered dressing	Mölnlycke	Mepilex Border
Polyurethane foam dressing	Dr. Ausbüttel & Co. GmbH	DracoFoam
Sticking plaster	Dr. Ausbüttel & Co. GmbH	DracoPor
Superabsorbent wound dressing	Absorbest AB	DryMax
Ointment	Ratiopharm GmbH	Iodine ointment
Ointment	Smith & Nephew	Iodosorb
Ointment	Pharmacy	Zinc paste

This systematic documentation provided a detailed overview of the wound's clinical status, crucial for evaluating its progression and informing the treatment approach. Furthermore, these data were integral to correlating clinical features with the collected wound odour samples, facilitating a more comprehensive analysis of wound healing dynamics and potential microbial involvement.

### 2.7 Statistics

All values were expressed as means ± standard deviations. Statistical analysis of all clinical parameters was performed using the software tools Microsoft Excel version 16.92 and GraphPad Prism version 9.5.1 (GraphPad Software, LLC) using One‐way ANOVA. The results of the VOCs detected by GC–IMS or the GC–MS are presented qualitatively and descriptively in terms of the frequency identified. The exportation of the measurements and the statistical analysis were conducted using Microsoft Excel.

## 3 RESULTS

### 3.1 Demographics and patient outcomes

**FIGURE 2:**
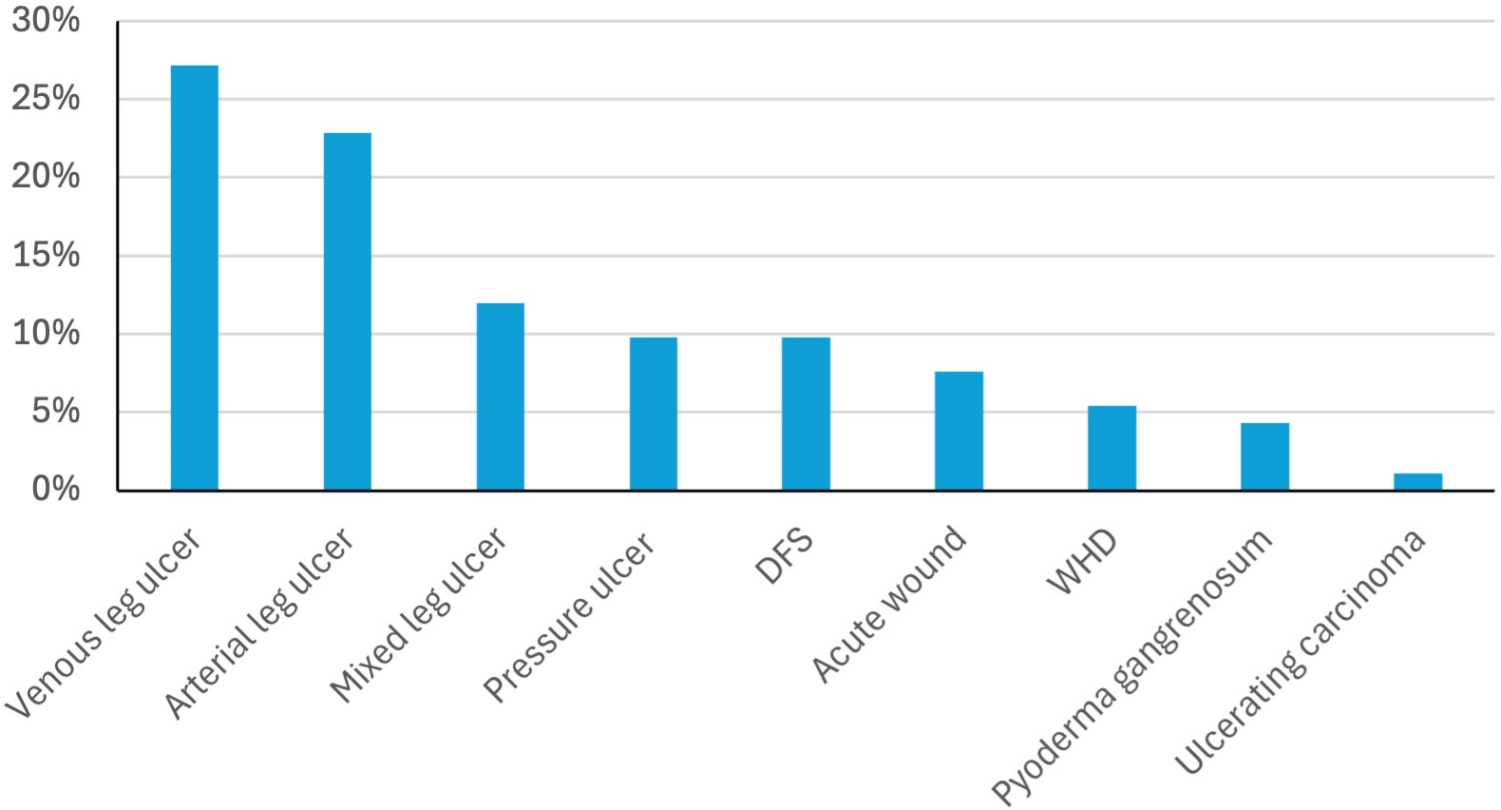
Frequency of different wound types, including diabetic foot syndrome (DFS) and wound healing disorder (WHD). Multiple selections per patient are possible.

**FIGURE 3:**
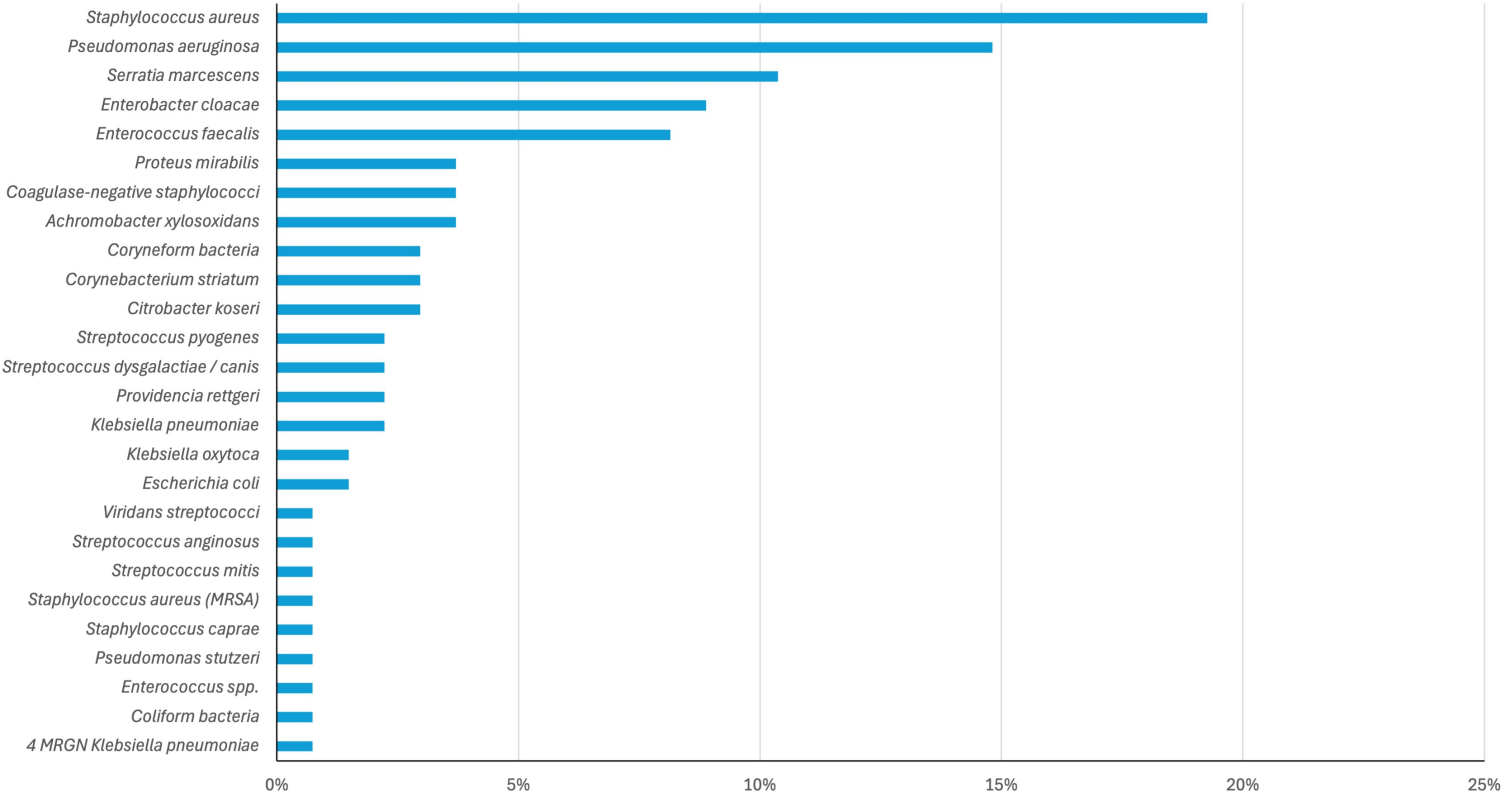
Frequency of detection of different bacterial species in the clinical microbiological routine swab results. Multiple selections per patient are possible.

Patients suffering from (chronic) wounds were recruited between January and September 2024. The study population included 26 women (39%) and 40 men (61%) with an average age of 69 ± 13 (IQR: 77–64) years and a body mass index (BMI) of 27.0 ± 4.8 (30.0–23.7) kg/m^2^. Persistence of chronic wounds averaged 24.3 ± 70.8 months (24‐4). Excluding outliers, the mean wound age was 12.5 ± 11.6 (18‐4) months. In total, we analysed 92 wounds from those 66 patients. The most common diagnoses included venous leg ulcer (27%), arterial leg ulcer (23%) and mixed leg ulcer (12%). Other diagnoses observed were diabetic foot syndrome (10%), pressure ulcers (10%), acute wounds (8%) and wound healing disorders (5%) (Figure 2). Less frequent conditions included pyoderma gangrenosum (4%) and rare cases of ulcerating carcinoma (1%). By swabbing, 135 pathogens were identified by culture in routine clinical microbiology (Figure 3). In relation to the bacterial species, 19% *Staphylococcus aureus*, 15% *P. aeruginosa* and 35% *Enterobacteria* were detected.

### 3.2 The outcome of patients' HRQoL

**FIGURE 4:**
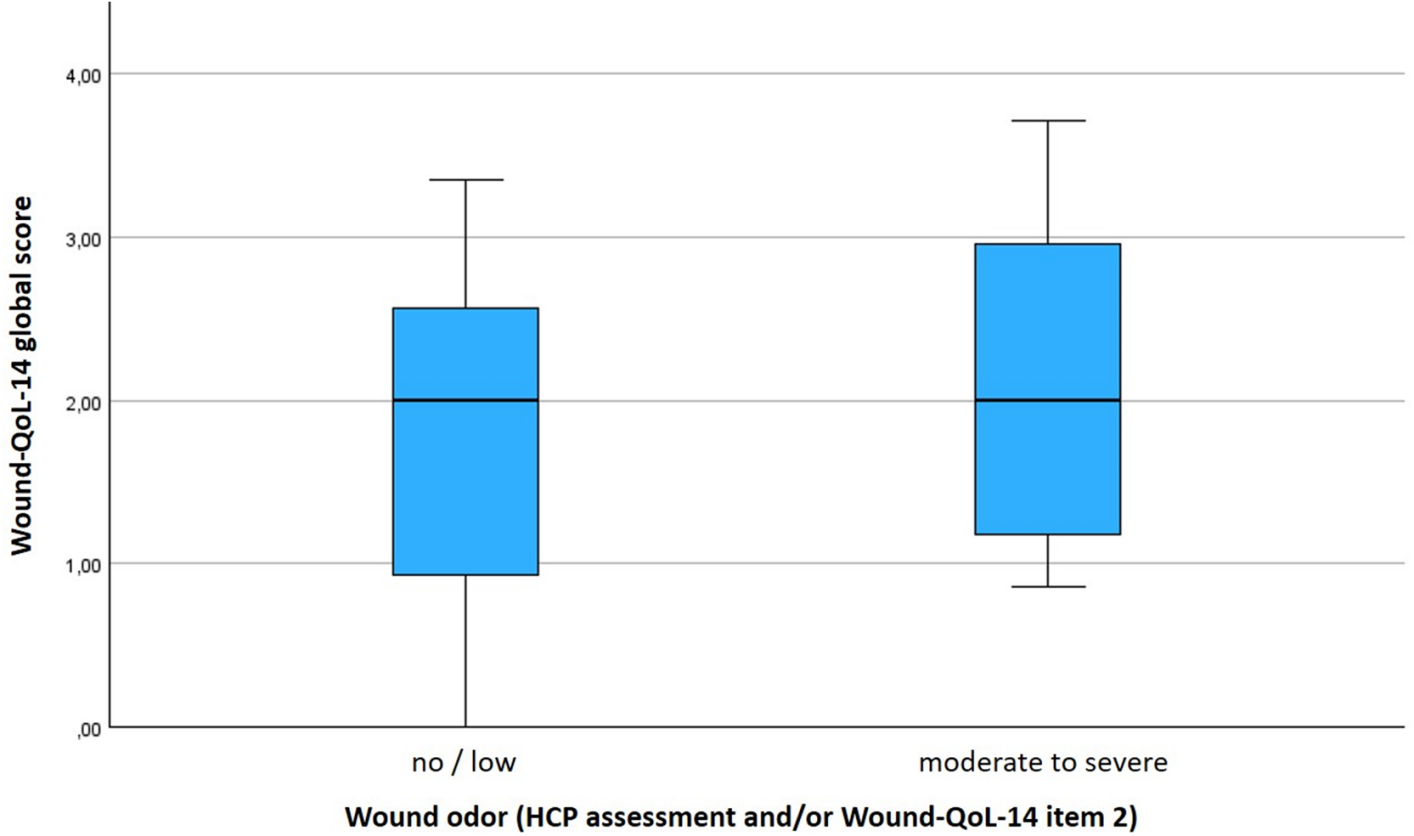
Boxplots illustrating HRQoL of 66 patients with 92 wounds, categorised by ‘no/low’ or ‘moderate to severe’ wound odour and determined in the Wound‐QoL‐14 questionnaire.

The Wound‐QoL‐14 mean score was 1.8 ± 1.0, ranging from 0.0 to 3.7. Patients classified as having severe wound odour (either due to odour‐related item in the Wound‐QoL‐14 or expert opinion) had slightly higher Wound‐QoL‐14 scores (i.e., higher impairments in HRQoL) than those classified as having no/low wound odour (Figure 4), which was not significant (2.1 ± 1.0 vs. 1.8 ± 1.0). This result is not statistically robust due to the significantly different number of patients included with and without wound odour (10:56 patients).

**FIGURE 5:**
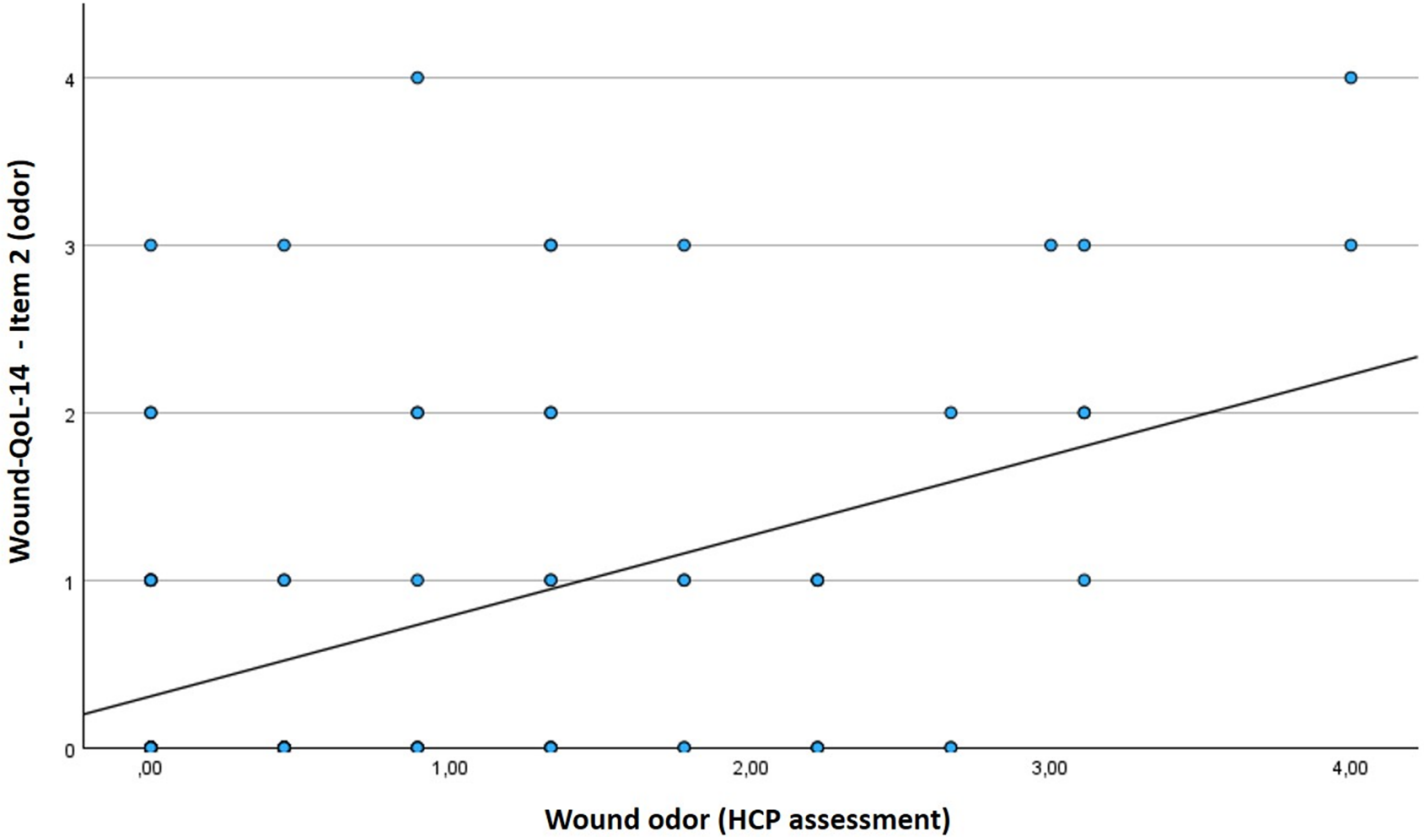
Correlation between the odour‐related item 2 in the questionnaire ‘Wound‐QoL‐14’ and the subjective HCP assessment of wound odour intensity on a scale from 0 to 4 (no to severe; *p* < 0.001).

HCPs' perception of malodour and the patients' assessment of their malodour were relatively synchronous. However, with regard to the parameter ‘no odour’ or ‘weak odour’, the subjective assessment varied for 18 wounds, which can be attributed to differences in individual olfactory perception. In sum, HCP assessment of wound odour showed no significant correlation with the Wound‐QoL‐14 total score (*r* = 0.000, *p* = 1.000), but a significant correlation with the odour‐related item in the Wound‐QoL‐14 (item 2; *r* = 0.385, *p* < 0.001; Figure 5).

### 3.3 Analysis of wound odour

#### 3.3.1 Gas chromatography–ion mobility spectrometry (GC–IMS)

**FIGURE 6:**
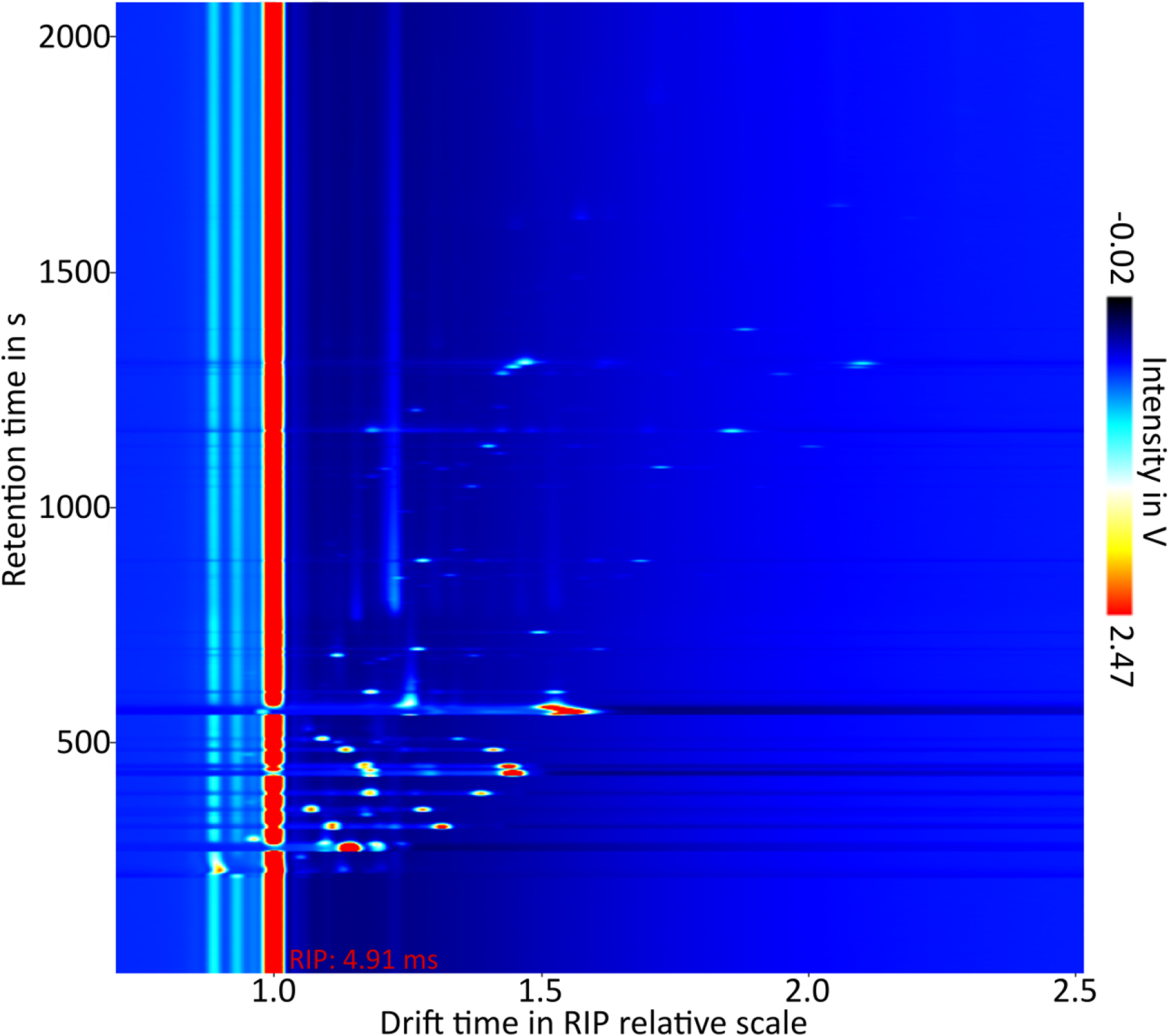
Example of a GC–IMS diagram from a wound dressing of a patient with an ischemic ulcer. The retention time in seconds is on the y‐axis and the drift time is on the x‐axis relative to the reactant ion peak (drift time 4.91 ms). The different colour spectrum refers to different signal intensities in V (blue: Low; red: High).

The GC–IMS analysis shows 191 signals from the wound samples. A GC–IMS chromatogram from a headspace‐sampled wound dressing is shown in Figure 6. The 10 most frequently detected VOCs are listed in Table 2. Five can be tentatively identified as butanone, hexanal, isopropyl acetate, 2‐pentanone and isoprenol. However, it is notable that substances with a known strong characteristic odour do not appear in the table but are also very common. For instance, diacetyl (2,3‐butanedione) was detected in 70 samples, while dimethyl disulphide was detected in 56 samples. Among the most frequently found substances are those that could not be definitively identified with the help of the database. These substances were assigned a numerical identifier for organisation and reference.

**TABLE 2:** Summary of the 10 substances most frequently detected by the GC–IMS, CAS#: Chemical abstract service number of a chemical compound.

Compound name	CAS#	Frequency
Unknown (1)	–	88
Unknown (4)	–	88
Unknown (8)	–	88
Butanone	78‐93‐3	84
Hexanal	66‐25‐1	79
Isopropyl acetate	108‐21‐4	78
Isoprenol	763‐32‐6	78
Unknown (6)	–	77
2‐Pentanone	107‐87‐9	75
Unknown (2)	–	75

The intensity of the detected VOCs varied, with dimethyl disulphide showing the highest average intensity among the tentatively identified compounds. Table 3 lists the 10 VOCs with the highest average intensity. In addition to dimethyl sulphide and five unknown compounds, cycloten, 1‐heptanol, phenol and 3‐hydroxy‐2‐butanone were found with high intensities.

**TABLE 3:** Summary of the 10 most frequently detected substances (odours) with the highest average intensity, CAS#: Chemical abstract service number of a chemical compound.

Compound name	CAS#	Average intensity (mV)
Unknown (1)	–	4.24
Dimethyl disulfide	624‐92‐0	3.77
Cyclotene	80‐71‐7	2.89
1‐Heptanol	111‐70‐6	2.60
Unknown (4)	–	2.13
Phenol	108‐95‐2	2.13
3‐Hydroxy‐2‐butanone	513‐86‐0	1.90
Unknown (2)	–	1.83
Unknown (44)	–	1.55
Unknown (9)	–	1.50

#### 3.3.2 Gas chromatography–mass spectrometry (GC–MS)

The GC–MS analysis has tentatively identified 183 different VOCs using the NIST database. However, it is important to emphasise that within the scope of this feasibility study, conducting reference measurements with standards for unequivocal identification was not planned. On average, 14 substances were detected per gas sample from the wound dressings. The most frequently identified compounds included dimethyl disulphide, 2,2‐dimethyldecane and ethylbenzene (Table 4).

**TABLE 4:** Most frequently detected VOCs in GC–MSanalysis, CAS#: Chemical abstract service number of a chemical compound.

Compound name	CAS#	Frequency
Dimethyl disulphide	624‐92‐0	25
2,2‐Dimethyldecane	17,302‐37‐3	16
Ethylbenzene	100‐41‐4	15
3‐Methyl‐1‐butanol	123‐51‐3	14
1‐Ethyl‐2‐methylbenzene	611‐14‐3	14
2‐Propyl‐1‐pentanol	58,175‐57‐8	13
Tetradecane	629‐59‐4	11
Propylbenzene	103‐65‐1	9
Butanone	78‐93‐3	8
Isovaleraldehyde	590‐86‐3	8

**FIGURE 7:**
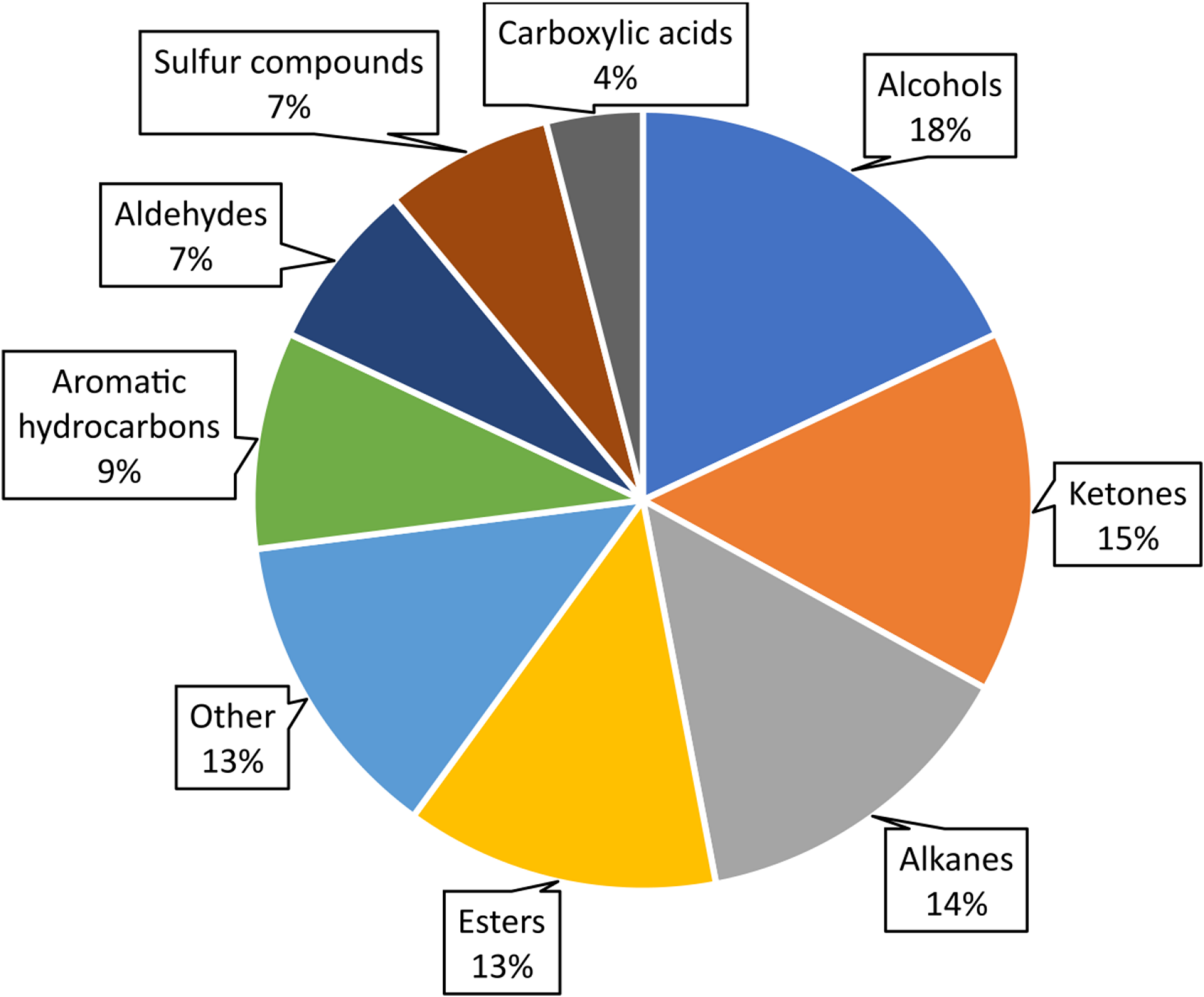
Substance group distribution of the VOCs in GC–MS analysis of wound dressings.

The analysis also revealed that primary alcohols, ketones and alkanes were the most frequently identified substance groups (Figure 7). The distribution is consistent with the results of the GC–IMS measurements, where these substance groups also dominated.

#### 3.3.3 Correlation of GC–IMS and GC–MS Results

The combination of the two analytical methods demonstrated that they were partially complementary in the identification of substances. After adjusting the retention times, correlations between the measurement results were possible. A notable outcome of this analysis was the identification of dimethyl disulphide, which was detected in both measurement methods and can, therefore, be considered unequivocally identified.

This approach enabled the correlation of 23 additional substances from the two analyses. This enhancement in the reliability of substance identification is attributable to the independence of detection by two different methods, which provides additional confirmation. The substance groups of alcohols and ketones exhibited particularly frequent correlations in the measurements carried out.

Especially for the GC–IMS, the parallel use of the MS is advantageous, as the IMS database has very few entries. The results of the parallel analysis suggest that the unknown substance no. 71 could be phenylethanol and no. 103 2,3‐heptanedione. It is not possible to correlate other unknown substances from the GC–IMS measurement, as these were either outside the retention time range of the reference substances for correlating via the retention index or the mass spectra did not match the database sufficiently.

## 4 DISCUSSION

Wound odour is a common problem for many patients with chronic and malignant wounds. Patients and healthcare professionals (HCPs) perceive wound odour as a nuisance. Unpleasant wound odour significantly impacts the patient's quality of life. Patients often show a tendency towards embarrassment, which can lead to social isolation.^4^ The impact of wound odour and its management is of significant importance: It has been documented in the scientific literature that tumour patients undergoing palliative care sometimes report finding wound odour more distressing than wound pain.^8^, ^18^

Malodour and the associated embarrassment of bothering others often lead to social distancing and reduced HRQoL. The standardised and internationally validated Wound‐QoL questionnaire on the quality of life of patients with chronic wounds addresses the topic of ‘wound odour’ with one of 14 questions.^14^, ^19^ The present results show a difference, although not significant (>0.5 in terms of clinical relevance), in the assessment of HRQoL in patients in whom the HCPs also rated the wound odour as worse or more intense. It can be postulated that this effect would be more pronounced if the group size of patients with and without malodour were not so different (10 vs. 52 patients). Further studies with at least 50% of the patients with malodour will confirm the importance of the ‘wound odour’ item in the HRQoL.

As artificial intelligence (AI) continues to develop and deeper insights into the composition of wound odour are gained, the idea of electronic nose (e‐nose) technology is also emerging.^20^ The goal is to use VOCs in wound odour to detect negative wound development or even the presence of a specific bacterial species. This idea is not entirely new and can be found in the literature. However, the original aim of this development was to generate military and industrial safety (e.g., leakage of hazardous chemicals)^21^ or to detect drug use (cannabis).^22^ There are preliminary advantages in medicine for diagnosing respiratory diseases or lung tumours.^23^, ^24^ Significant differences in VOCs have also been successfully detected in the differentiation between infected and non‐infected burns using swabs and dressings.^25^ The analysis of wound odour by e‐nose may provide faster results regarding the type and intensity of bacterial load and thus allow early antiseptic therapy, but it does not replace an antibiogram. In addition, these devices lack the olfactory interpretation of the human brain, which distinguishes more than a trillion different odours into ‘pleasant’ and ‘unpleasant’ odours on an individual basis.^26^

A key approach to reducing wound odour, often forgotten in the focus on local therapy, is managing the underlying disease. Appropriate management of diabetes mellitus or vascular intervention for PAD or CVI is essential for wound healing and ensures local immune competence to combat bacterial burden, biofilm or infection. This prevents necrosis, the (bacterial) breakdown of which is a major contributor to wound malodour. On the other hand, studies show that systemic and local infection control measures reduce the amount of VOCs.^27^ Using topical metronidazole, which is not approved in Europe or by the FDA, is particularly promising as previous studies demonstrated a reduction in anaerobic bacteria in wounds and, consequently, wound odour.^28^ In off‐label use, mostly in palliative care, it is regularly used for exuding tumour wounds.^18^, ^29^, ^30^

There is a wide range of wound dressings on the market today, most of which are based on activated charcoal. However, only 48.4% of HCPs report a sufficient effect on odour reduction.^8^ For this reason, an increasing number of in vitro studies (mostly using pathogenic bacteria and the VOCs of their metabolites) are concerned with the identification, analysis and possible ‘therapy’ or masking of the substances that are unpleasant to human olfactory perception.^31^, ^32^ To date, however, none of these studies have yet reached the stage of clinical application. In situations where patients, family members and clinicians have failed in their efforts, alternative measures are used. Various household remedies such as coffee grounds, cat litter, essential oils and air fresheners have limited effectiveness.^8^, ^18^

Current clinical options using antibacterial dressings containing silver,^33^, ^34^ polyhexamethylene biguanide (PHMB)^35^ or octenidine^36^ are limited in their ability to absorb wound odour; they cannot eliminate VOCs resulting from tissue degeneration such as cell death or necrosis, and bacterial reduction takes time, so unpleasant wound odour persists in the interim.^30^ This highlights the need for alternative and more effective solutions.

VOCs are the primary cause of wound odour.^37^ In the context of wound odour, these include diamines such as cadaverine or putrescine, also known as cadaveric toxins.^4^, ^38^ They also include short‐chain fatty acids (e.g., butyric acid) or sulphide compounds (e.g., dimethyl trisulphide).^39^ Tissue degeneration or bacterial colonisation of the wound is often cited as a source of wound odour.^40^, ^41^

GC–MS and GC–IMS are generally able to detect VOCs emitted from the wounds. In previous studies, this has been shown, for example, by direct gas sampling^42^ or by analysing the headspace of bacteria cultures from the wounds.^25^ However, the crucial point of any analytical method is the sampling step. The shown feasibility study uses headspace sampling of the wound dressing, which makes sampling easier, leading to the possibility of a higher sample size and more controlled conditions. The used system is more capable than mobile devices and has much potential for identifying many VOCs.

The chemical analyses show that the wound odour cannot be attributed to a single substance but is caused by many VOCs. After removing background information and blanks from the measurement data, the exact origin of these substances remains unclear. Some identified VOCs do not appear to originate directly from the wound but from external sources such as cosmetics or cleaning agents used by patients or HCPs. Examples include rose oxide, α‐pinene and p‐cymene, which are widely used in cosmetic products.^43^, ^44^, ^45^ Acetic acid isopropyl ester, a substance used in the food industry as both a flavouring agent and a solvent, was also detected, although a microbial source for this substance cannot be ruled out but is considered unlikely.^46^, ^47^

Another group of VOCs can be attributed to bacterial metabolism. Ketones such as butanone, pentanone and heptanone are known metabolites of *P. aeruginosa* ^48^ Alcohols, including propanol and butanol, also occur as intermediates in bacterial metabolism.^49^ Ethanol and isopropanol may originate from similar processes, but in the clinical setting, their origin from hand rubs is more likely.^50^ Isoprene, which is produced by a variety of microbial pathogens as well as in human cholesterol metabolism, occupies a special position.^51^, ^52^ Since humans exhale isoprene, it is difficult to verify its exact source in measurements.^53^ On the other hand, typical bacterial ‘biomarkers’ have been identified that can be assigned to specific species: Dimethyl trisulphide, typical of *P. aeruginosa* or indole, which is often associated with *Escherichia coli* and serves as a biomarker for certain *E. coli* strains in biofilms.^54^, ^55^ An accumulation of these VOCs has been demonstrated in fungating cancer wounds.^39^

In addition, the sulphide compounds detected, such as dimethyl disulphide and dimethyl trisulphide, are likely indicators of inflammatory processes. Animal models have already demonstrated an association between sulphides and inflammation.^27^ However, there was no correlation between the presence of sulphur compounds and clinical signs of inflammation. Interestingly, some VOCs previously described in the literature to be associated with wound odour were not detected: Caproic, caprylic and isovaleric acids, and the diamines cadaverine and putrescine.^4^, ^56^ However, this may be because these results were detected in burn wounds, considered acute or acutely infected, rather than chronic wounds as in this project. This might possibly be explained by the fact that this is a preliminary exploratory study and the measurement method needs to be further optimised.

A comparison of wound characteristics revealed differences in wound odour perception. Wounds with biofilm burden had an average higher odour perception of 3.8 (on a scale of 0–10) without a significant increase in the number of substances detected. In contrast, wounds with incipient epithelialisation had significantly lower odour perception (1.8). On the other hand, chemical analysis of the latter showed a lower number of VOCs and a clinical absence of signs of inflammation. These observations conclude that the number of VOCs does not necessarily correlate with the intensity of the perceived odour; the quality (e.g., sulphide compounds) rather than the quantity of VOCs is important. Knowing which VOCs are responsible for the negative wound odours that humans find unpleasant, it may be possible to identify causal ‘antagonist’ chemical compounds that mask these odours to human olfactory perception. One example is Timberol®, which can mask the very unpleasant smell of fish based on trimethylamine (TMA) by targeting/blocking its odourant receptor.^57^

Despite some positive findings regarding the potential development of new treatment strategies to reduce malodour, this exploratory single‐centre study has several limitations. The analysis of the broad spectrum of wounds and wound patients does not differentiate based on underlying conditions, nor does it take into account individual bacterial colonisation, as the respective ‘group size’ would be too small. The microbiological burden of the wounds was determined using standard microbiological routines on typical culture media, which limits the range of bacterial species detected.^58^ If the swabs had been analysed using polymerase chain reaction (PCR), a method not yet part of routine clinical practice, it is likely that a greater variety of additional bacterial species would have been identified in the wounds.^59^ The currently routinely employed techniques lack the ability to detect anaerobic bacteria, which precluded any correlations in this regard.^60^ However, a significant proportion of the intensity of the perceived wound odour can certainly be attributed to these anaerobic bacteria. Future studies should include a greater number of fungating wounds to better incorporate patients who would benefit most from the development of new anti‐malodour strategies. The detection of typical VOCs in the malodour of fungating wounds is insufficient in this exploratory study. In comparison to current, partially successful local malodour treatment strategies, which typically work indirectly by targeting bacterial load (e.g., metronidazole)^61^ or through absorption (e.g., activated charcoal),^7^ analysing VOCs presents an opportunity to address odour directly in the near future.

## 5 CONCLUSIONS

Chronic wounds are colonised by various bacteria, which, together with tissue damage and necrosis, are responsible for the development of wound odour. HRQoL analysed with the questionnaire Wound‐QoL‐14 showed a trend towards reduction in patients with wound odour, although the size of the groups with wound odour compared to no/low wound odour was different (10 vs. 82 patients). The GC–MS as well as the GC–IMS analyses provided valuable insights into the composition of volatile organic compounds (VOCs) from wound samples and demonstrated that this method is a reliable analytical tool. In particular, the technical ability to identify, from the broad spectrum of VOCs present in wound odour, those that most significantly impact patients and healthcare providers—and potentially even induce feelings of disgust—could represent an innovative approach for the targeted development of new anti‐odour strategies in wound management. The aim would be to not only neutralise or absorb odours but also selectively inhibit the VOCs responsible for malodour, preventing their detection by humans. A more detailed chemical analysis will show whether the typical odours of the bacteria detected in the wound swab by PCR can be identified or filtered out from the broad spectrum of VOCs.

## AUTHOR CONTRIBUTIONS

*Conceptualisation*: E. K. Stuermer, M. Augustin and S. C. Liegenfeld. *Data curation*: H. Lober, C. Nathrath, M. Dittmer and T. M. Janke. *Investigation*: H. Lober, M. Wigger, C. Nathrath, M. Dittmer and S. C. Liegenfeld. *Methodology*: H. Lober, M. Wigger, C. Nathrath and T. M. Janke. *Project administration*: E. K. Stuermer, M. Augustin and S. C. Liegenfeld. *Resources*: E. K. Stuermer, S. Sielemann and M. Augustin. *Supervision*: E. K. Stuermer, S. Sielemann and M. Augustin. *Validation*: E. K. Stuermer. *Visualisation*: H. Lober, C. Nathrath, T. M. Janke and M. Dittmer. *Writing – original draft*: E. K. Stuermer, H. Lober and T. M. Janke. *Writing – review and editing*: E. K. Stuermer, H. Lober, M. Wigger and S. Sielemann.

## CONFLICT OF INTEREST STATEMENT

The authors declare no conflicts of interest with regard to this project. E. K. Stuermer received fees for consulting, lectures and/or studies from Institut AllergoSan, BEMER Inc., Curea medical, DEBx medical, essity, Schülke and Mayr, Serag‐Wiessner and Smith and Nephew. M. Augustin has served as consultant and/or paid speaker for and/or has received research grants and/or honoraries for consulting and/or scientific lectures for and/or got travel expenses reimbursed and/or participated in clinical trials sponsored by companies that manufacture drugs used for the treatment of wounds including 3M, AOK Bundesverband, Bayer Healthcare, Beiersdorf, Birken, BSN, BVmed, Coloplast, DAK, Diabet concept, Mölnlycke, Smith&Nephew, Schülke&Mayr, Söring and Urgo. Henrik Lober is currently employed as an intern at Dr. Ausbüttel & Co. GmbH, Dortmund, Germany.

## Data Availability

The data that support the findings of this study are available from the corresponding author upon reasonable request.
